# Analysis of GABAergic and Non-GABAergic Neuron Activity in the Optic Lobes of the Forager and Re-Orienting Worker Honeybee (*Apis mellifera* L.)

**DOI:** 10.1371/journal.pone.0008833

**Published:** 2010-01-21

**Authors:** Taketoshi Kiya, Takeo Kubo

**Affiliations:** Department of Biological Sciences, Graduate School of Science, The University of Tokyo, Tokyo, Japan; Centre de Recherches su la Cognition Animale - Centre National de la Recherche Scientifique and Université Paul Sabatier, France

## Abstract

**Background:**

European honeybee (*Apis mellifera* L.) foragers have a highly developed visual system that is used for navigation. To clarify the neural basis underlying the highly sophisticated visual ability of foragers, we investigated the neural activity pattern of the optic lobes (OLs) in pollen-foragers and re-orienting bees, using the immediate early gene *kakusei* as a neural activity marker.

**Methodology/Principal Findings:**

We performed double-*in situ* hybridization of *kakusei* and *Amgad*, the honeybee homolog of the GABA synthesizing enzyme GAD, to assess inhibitory neural activity. *kakusei*-related activity in GABAergic and non-GABAergic neurons was strongly upregulated in the OLs of the foragers and re-orienting bees, suggesting that both types of neurons are involved in visual information processing. GABAergic neuron activity was significantly higher than non-GABAergic neuron activity in a part of the OLs of only the forager, suggesting that unique information processing occurs in the OLs of foragers. In contrast, GABAergic neuron activity in the antennal lobe was significantly lower than that of GABAergic neurons in the OLs in the forager and re-orienting bees, suggesting that *kakusei*-related visual activity is dominant in the brains of these bees.

**Conclusions/Significance:**

The present study provides the first evidence that GABAergic neurons are highly active in the OL neurons of free-moving honeybees and essential clue to reveal neural basis of the sophisticated visual ability that is equipped in the small and simple brain.

## Introduction

European honeybee (*Apis mellifera* L.) workers forage for food sources using their highly developed visual sense [1]–[3]. After returning from foraging flights, successful foragers transmit information about the sites of rich food sources to their nest mates using dance communication [2], [4]. Foragers gauge the distance of the food source based on the amount of optic flow received during their flight [1], [3]. Although it is well know that foragers have highly sophisticated visual ability, the neural basis of the visual information processing associated with the foraging behavior remains unknown.

The honeybee brain comprises several distinct regions, including the mushroom bodies (MBs), a higher-order integration center; the optic lobes (OLs), a visual center; and the antennal lobes (ALs), the olfactory center [5], [6]. The MBs are mainly composed of two morphologically distinct types of interneurons, termed large-type and small-type Kenyon cells [6].

We previously identified a novel immediate early gene, *kakusei*, that can be used as a marker of neural activity, and showed that the neural activity of the small-type Kenyon cells is preferentially increased in the forager brain [7]. We also detected *kakusei* expression in other areas of the forager brain, including the OLs. Due to the lack of appropriate criteria to discriminate cell types, however, only gross *kakusei*-positive cell numbers in these brain regions could be counted.

To elucidate the neural basis of the sophisticated visual ability of the foragers, clarification of the neural activity pattern in the OLs of the foragers is essential. In the present study, to examine neural activity in the OL neurons in detail, we focused on γ-aminobutyric acid (GABA) neurons to discriminate between excitatory and inhibitory neural activity. Not only GABA but also histamine and hyperpolarizing glutamate function as inhibitory neurotransmitters in the insect brains [8]–[10], although we focused only on GABA in the present study.

GABA is the major neurotransmitter for inhibitory synapses in both the vertebrate and invertebrate nervous systems [11]. In the honeybee brain, GABA neurons are widely distributed to the whole brain area [12]–[14], and play important roles in the sensory processing (e.g., olfaction) and sensory integration (e.g., olfactory learning) [15]–[17]. Thus, in addition to their functional importance, GABAergic neurons can be an appropriate marker to investigate inhibitory neural activity in the forager brain.

To investigate GABAergic neuron activity, we performed double *in situ* hybridization to simultaneously detect the expression of *kakusei* and *Amgad*, the honeybee homolog of the gene for the GABA synthesizing enzyme GAD, as a marker for GABAergic neurons. The *kakusei*-related activity of both GABAergic and non-GABAergic neurons was upregulated in the OLs in the pollen forager and re-orienting bees. GABAergic neuron activity was significantly increased in a part of the OLs of only the forager, suggesting that unique information processing occurs in the forager OLs. In contrast, GABAergic neuron activity in the AL was low in the forager and re-orienting bees, suggesting that olfactory activity is not high and visual activity is dominant in these bees. This is the first report showing that GABAergic neuron activity is highly increased in free-moving honeybees.

## Materials and Methods

### Bees

European honeybees (*A. mellifera* L.) were purchased from a local dealer (Kumagaya Honeybee Farm, Saitama, Japan) and maintained at the University of Tokyo. Workers were collected using different methods depending on the experimental purpose. In this paper, we investigated the number of *kakusei*-expressing cells in the brains of seizure-induced bees, foragers, re-orientation bees, and light-exposed bees. For the foragers (n = 9), workers that returned to the hive with pollen loads were caught in front of the hive entrances. To maintain the current state of gene expression in the brains, the bees were caught and immediately immersed in ice-cold water and kept on ice until use for *in situ* hybridization. For the seizure-induced bees (n = 6), foragers whose wings were cut were kept in the cage overnight at 30°C. The next day, the bees were anesthetized with CO_2_ for 5 min and then awakened from the anesthesia by exposure to normal air. At the time of awakening from CO_2_-induced anesthesia, the bees shook their legs and showed a seizure-like phenotype [7], [18]. To fully induce *kakusei* expression, the bees were kept at 30°C for 30 min after awakening and then used for *in situ* hybridization. For the re-orienting bees, the hive location was moved at night with the entrance closed and the next morning the entrance was opened for 5 min and the bees were caught while they were flying around the hive 0 and 15 min later (n's = 4 and 6, respectively). For the light-exposed bees, foragers whose wings were cut were kept in a dark incubator overnight at 25°C [dark-adapted bees, n = 8]. The next day, the bees were exposed to white light for 30 min at 25°C, and then used for *in situ* hybridization [light-exposed bees, n = 10].

### cDNA Cloning

To isolate *Amgad* DNA fragments, total RNA was isolated from the brains of workers with TRIzol (Invitrogen, Carlsbad, CA), treated with DNase I (Invitrogen), and reverse-transcribed with SuperScript II First-Strand Synthesis System (Invitrogen). Polymerase chain reaction (PCR) was performed with *ExTaq* polymerase (Takara, Tokyo, Japan) using gene-specific primers for *Amgad* (5′-AATGGTGAACGTCTGCTTCTGGTAT-3′ and 5′-ACTTACGTGCTATGAGTATCCTTTG-3′), producing an approximately 0.8-kbp fragment. The PCR products were subcloned into pGEM-T vectors (Promega, Madison, WI), and sequenced to confirm that *Amgad* was successfully isolated. Experiments were performed according to the manufacturers' protocols. Accession number of predicted *Amgad* and *kakusei* is XM_391979 and AB252862, respectively. The protein sequence similarity of full-length GAD between species was calculated using the DNASIS Pro software (Hitachi Software Engineering, Tokyo, Japan) with the default setting.

### *In situ* Hybridization

Brains of bees were dissected out, frozen in OTC Tissue-Tek compound (Sakura Fine Technical, Tokyo, Japan) on dry ice, and stored at −80°C until use. Frozen coronal brain sections (10-µm thick) were cut onto 3-aminopropyltri-ethoxysilane-coated glass slides (Matsunami, Tokyo, Japan). Slides were air-dried and stored frozen at −20°C until use.

Biotin-labeled *kakusei* riboprobes were synthesized by T7 or SP6 polymerase with a biotin RNA labeling mix (Roche, Switzerland) using a plasmid containing *kakusei* cDNA as previously described [7]. Digoxigenin (DIG)-labeled *Amgad* riboprobes were synthesized with a DIG RNA labeling mix (Roche) using a plasmid containing an approximately 0.8-kbp cDNA isolated by RT-PCR. The probe templates were prepared by PCR using SP6 and T7 primers.

The sections were fixed in 4% paraformaldehyde in phosphate buffer (PB; pH 7.4) overnight at 4°C, treated with proteinase K (10 µg/ml) for 15 min and then with HCl (0.2 N) for 10 min, followed by acetic-anhydride solution for 10 min at room temperature. The slides were washed with PB between each step. After dehydration through serial ethanol solutions, brain sections were hybridized with DIG-labeled *Amgad* riboprobes at 60°C overnight (>16 h). The riboprobes were diluted in hybridization buffer (50% formamide, 10 mM Tris-HCl, 200 µg/ml tRNA, 1×Denhardt's solution, 10% dextran sulphate, 600 mM NaCl, 0.25% SDS, 1 mM EDTA at pH 7.6), heat-denatured at 85°C for 10 min, and then added to each slide. After hybridization, slides were washed in 50% formamide and 2×SSC at 60°C for 30 min, treated with 10 µg/ml RNase A (Sigma-Aldrich, St. Louis, MO) in TNE (10 mM Tris-HCl, 1 mM EDTA, 500 mM NaCl at pH 7.6) at 37°C for 30 min, and washed at 60°C twice in 2×SSC for 20 min and 0.2×SSC for 20 min. DIG-labeled riboprobes were detected immunocytochemically with alkaline phosphatase-conjugated anti-DIG antibody and 5-bromo-4-chloro-3′-indolyphosphate p-toluidine salt and nitro-blue tetrazolium chloride using a DIG nucleic acid detection kit (Roche) according to the manufacturer's protocol.

For combined detection of *kakusei* and *Amgad* by fluorescent *in situ* hybridization, a mixture of biotin-labeled *kakusei* and DIG-labeled *Amgad* riboprobes was added during the hybridization step. Following the series of washes described above and blocking with the reagent from the TSA Biotin System (Perkin Elmer, Norwalk, CT), the two differentially labeled probes were detected sequentially. First, to detect the biotin-labeled *kakusei* probes, slides were incubated with streptavidin horseradish-peroxidase (HRP) conjugate (1∶1000, Perkin Elmer) at 37°C for 1 h, tyramide-biotin working solution (Perkin Elmer) at 37°C for 10 min, and streptavidin alkaline-phosphatase conjugated (1∶1000, Vector Laboratories, Burlingame, CA) at 37°C for 45 min, and washed three times in Tris-buffered saline (with 0.05% Tween-20) between each step. Following a wash with detection buffer (100 mM Tris-HCl, 100 mM NaCl, 10 mM MgCl_2_ at pH 8.0) for 5 min, the slides were incubated with an HNPP/Fast Red TR mix (Roche) at room temperature for 30 min. The slides were then treated with 1% H_2_O_2_ for 15 min to quench the residual HRP activity. To detect the DIG-labeled *Amgad* probes, the slides were then incubated with anti-DIG HRP (1∶500, Roche) at 37°C for 45 min, and tyramide-fluorescein working solution (Perkin Elmer) at 37°C for 10 min. After counterstaining with 30 nM DAPI (Invitrogen), the slides were coverslipped with SlowFade Gold antifade reagent (Invitrogen). Sense probes were used as negative controls and the signals were confirmed to be antisense probe-specific in every experiment. Intensity and brightness of the micrographs were processed with Photoshop software (Adobe Systems, San Jose, CA). The excitation/emission wavelengths for DAPI, fluorescein, and HNPP/Fast Red TR mix are 358 nm/461 nm, 494 nm/521 nm, and 553 nm/584 nm.

### Image Analysis and Cell Counting

Fluorescent *in situ* hybridization images were acquired using an Axio Imager.Z1m (Carl Zeiss, Germany) equipped with AxioCam HRm CCD camera. Optical sections (0.5 µm thick) were acquired with 100× oil-immersion objective lens using Apotome (Carl Zeiss), adjusting the settings to optimize for each section. The filters of the microscope were filter set 49 (Ex365, Em445/50), 38HE (Ex470/40, Em525/50), and 43HE (Ex550/25, Em605/70) (Carl Zeiss). The light source was extra high pressure mercury lamp (HBO103w, Carl Zeiss). Optical section images were collected from one to five sections for each brain region of each bee type, and stored for off-line analysis. After intensity and brightness adjustment with Photoshop software (Adobe), each image was analyzed using ImageJ analysis software (NIH, http://rsb.info.nih.gov/ij) with the cell counter plug-in. First, an RGB image was split into three channels, and then the number of nuclei stained by DAPI was counted as the number of cells. Together, using the RGB image, the number of cells that were positive for *kakusei* and *Amgad* was counted. Thus, cells were classified as (1) negative, neither *kakusei* nor *Amgad*; (2) *kakusei* only, one or two intense nuclear foci present; (3) *Amgad* only, cytoplasmic staining surrounding the nucleus; (4) double-positive for *kakusei* and *Amgad*. The number of each class of cells and the total number of cells were calculated by adding up every section for each bee. Using these factors, we calculated the percentage of *kakusei*-positive cells that were either *Amgad* (+) or -negative (−) for each bee. The numbers of analyzed bees, sections, and cells are summarized in Table S1.

### Statistical Analysis

Statistical analyses were conducted using JMP (SAS Institute, Cary, NC) and Excel-Toukei 2006 (SSRI, Japan) software. For comparisons between two groups, a two-tailed *Student*'s *t*-test was conducted. If the *F*-test revealed that the group variances were significantly different, *Welch's t*-test was used in place of *Student*'s *t*-test. For pairwise comparison, a two-tailed paired *t*-test was conducted. For comparisons among more than three groups, *Kruskal-Wallis* test was used in place of the usual analysis of variance (ANOVA), because *Bartlett's* test revealed that the group variances were significantly different in all such cases. When the *Kruskal-Wallis* test was significant, intragroup comparisons were conducted with *Man-Whitney's U* test. For group comparisons of two factors, a two-way ANOVA was conducted. A P value less than 0.05 was regarded as significant. All data are presented as the mean±standard error.

## Results

### Expression Analysis of *Amgad*, a GABAergic Neuron Marker, in the Worker Brain

To visualize GABAergic neurons in the honeybee brain, we first isolated the honeybee homologue of glutamic acid decarboxylase (*gad*), which catalyzes the formation of GABA from glutamate [11]. To isolate honeybee *gad* (*Amgad*; *Apis mellifera gad*), we searched for honeybee brain-expressed sequence tags (Honey Bee Brain EST Project) and obtained a contig sequence (contig 196) [19]. Analysis using the BeeBase revealed that contig 196 overlaps with *GB19979*. The predicted protein GB19979 showed high sequence similarities to *Drosophila* GAD1 (X76198.1) and mouse GAD67 (AAH27059), throughout the full-length sequence (76.7% and 47.7%, respectively). Motif search using the Pfam program (http://motif.genome.jp/) showed that the pyridoxal-dependent decarboxylase domain, which is essential catalytic region, is conserved in GB19979, strongly suggesting that GB19979 functions as a decarboxylase. Although there are two types of GAD (GAD1 and 2) in the insect nervous system, GB19979 had the highest sequence similarity with GAD1, which has a predominant role in the *Drosophila* brain [20]–[22]. In addition, *Amgad* was a single-copy gene in the honeybee genome. These results suggest that *GB19979* is the honeybee *gad* (*Amgad*).

**Figure 1:**
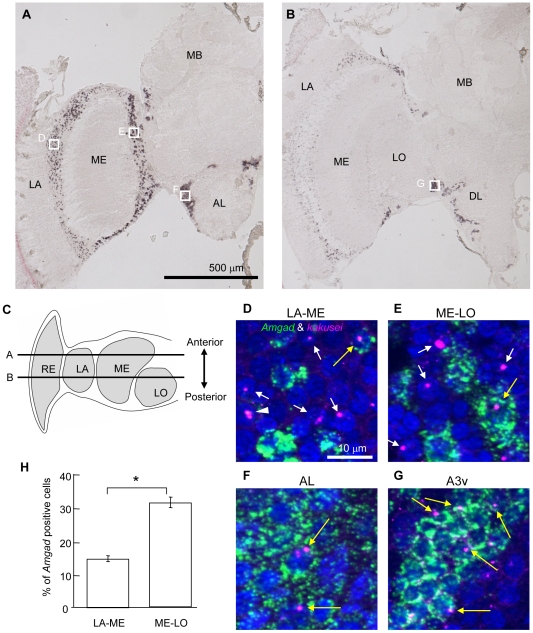
*Amgad* and *kakusei* expression in the worker brain. **A, B.** Expression of *Amgad* was detected by *in situ* hybridization using the rostral (**A**) and caudal (**B**) worker brain sections. Left hemispheres of coronal sections are shown. Note the strong *Amgad* signals in the optic lobe and antennal lobe neurons. **C.** Schematic drawing of the optic lobe of the worker brain and the position of the rostral (**A**) and caudal (**B**) sections. Dorsal view. Anterior is top. **D–G.** Double fluorescent *in situ* hybridization of *kakusei* (magenta) and *Amgad* (green) in the seizure (sz)-induced worker brain. The nuclei stained with DAPI are shown in blue. White arrows indicate *kakusei* (+) and *Amgad* (−) nuclei, and yellow arrows indicate *kakusei* (+) and *Amgad* (+) nuclei. Sometimes, nuclei with two intranuclear foci for *kakusei* were observed (white arrow head). The positions of (**D**)–(**G**) are indicated by the white squares in (**A**) and (**B**). **H.** The proportion of *Amgad* (+) cells was different between LA-ME and ME-LO. *: *P*<0.0001, *Welch's t*-test. Abbreviations: AL, antennal lobe; DL, dorsal lobe; LA, lamina; LO, lobula; ME, medulla; MB, mushroom body; RE, retina.

Next, to examine whether *Amgad* can be used as a marker of GABAergic neurons, we performed *in situ* hybridization of *Amgad*. *Amgad* expression was detected in the whole cortex of the OLs and ALs (Fig. 1A and B). In contrast, no significant signal was detected in the MBs (Fig. 1A–C). This staining pattern was consistent with the distribution pattern of GABA-like immunoreactivity reported previously [12], [13], indicating that *Amgad* can be used as a marker for GABAergic neurons in the honeybee brain.

### *kakusei* Is Expressed in GABAergic Neurons in the Worker Brain

Next, because we intended to use the immediate early gene, *kakusei*, to assess the *kakusei-*related activity of GABAergic neurons, we first examined whether *kakusei* was expressed in GABAergic neurons. The OLs consist of three layer structures: lamina, medulla, and lobula, from distal to proximal. Based on the *Amgad* expression pattern in the worker brain, in which the majority of GABAergic neurons were detected in the OLs and ALs (Fig. 1A and B), we focused our analysis on the following four regions: cells located between the lamina and medulla (LA-ME), medulla and lobula (ME-LO), lateral side of the AL (AL), and in the ventral root of the OL (A3v) (Fig. 1A and B). We selected LA-ME and ME-LO, in which both the morphology and projection pattern of the neurons have been well investigated [23]–[25], to assess the *kakusei*-related activity of GABAergic neurons in the OLs. Because these brain regions contained both *Amgad*–expressing [*Amgad* (+)] and *Amgad*-non-expressing [*Amgad* (−)] neurons, we counted the numbers of these two types of neurons to compare the *kakusei*-positive ratio. We selected a part of the AL region (AL) to assess the *kakusei*-related activity of GABAergic neurons in the ALs. We also selected A3v, which is a GABAergic neuron cluster that receives input from the MB α lobe and provides feedback input to the MB calyx, constituting a recurrent circuit [12], [14], [26], to assess the MB neuron activity from *kakusei*-related neural activity in A3v. As for AL and A3v, we counted *kakusei*-positive cells only in *Amgad* (+) cells, as both of these regions contained only *Amgad* (+) cells.

We then performed double *in situ* hybridization of *kakusei* and *Amgad* in seizure-induced worker brains to examine whether *kakusei* can be used to assess the GABAergic neuron activity in the worker brain. Seizures that are induced by awakening workers from CO_2_-induced anesthesia strongly induce *kakusei*-expression in the whole worker brain area [7]. Expression of *kakusei* was detected both in the *Amgad* (+) (yellow arrow) and (−) (white arrow) cells in all of LA-ME, ME-LO, AL, and A3v (Fig. 1D–G), strongly suggesting that *kakusei* can be used as an activity marker in GABAergic neurons. On one hand, there were also *Amgad* (+) cells with no significant *kakuse*i-expression in all four of these regions, possibly due to the lower *kakusei*-related activity in these neurons.

A previous study estimated that the number of GABA-like immunoreactive somata in the OLs is less than 5% [12]. In the present study, however, we detected a much higher number of *Amgad* (+) somata in the OLs; ca. 15% in LA-ME, ca. 30% in ME-LO. In addition, ME-LO had a significantly higher percentage of *Amgad* (+) cells than LA-ME (Fig. 1H). This discrepancy may be due to differences in sensitivity and specificity of the experimental procedures used.

**Figure 2:**
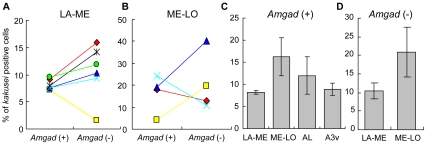
Percentage of *kakusei*-positive *Amgad* (+) and (−) cells in the seizure (sz)-induced bees. The data from each bee is shown by the same symbol and connected by a line. **A**, **B**. Percentage of *kakusei*-positive cells in LA-ME (**A**) and ME-LO (**B**). There was no significant difference in the *kakusei* expression between *Amgad* (+) and (−) cells in either region. **C**, **D**. Comparison of the percentages of *kakusei*-positive *Amgad* (+) (**C**) and (−) cells in various brain regions (**D**). No significant difference in the *kakusei* expression was detected among these brain regions.

We calculated the percentages of *kakusei*-positive *Amgad* (+) and (−) cells in LA-ME and ME-LO (Fig. 2A and B), and conducted a pairwise comparison of the percentage of *kakusei*-positive *Amgad* (+) and (−) cells for each worker. There was no significant difference in the percentage of *kakusei*-positive *Amgad* (+) and (−) cells in LA-ME or ME-LO. In addition, although *kakusei* expression tended to be higher in both *Amgad* (+) and (−) cells in ME-LO than in *Amgad* (+) and (−) cells in LA-ME, the percentage of *kakusei*-positive cells was not significantly different among brain regions, irrespective of *Amgad* expression (Fig. 2C and D). These results suggest that *kakusei* is expressed in various brain regions of seizure-induced workers in both GABAergic and non-GABAergic neurons.

### *kakusei*-Related Activity of GABAergic Neurons in the Forager Brain

**Figure 3:**
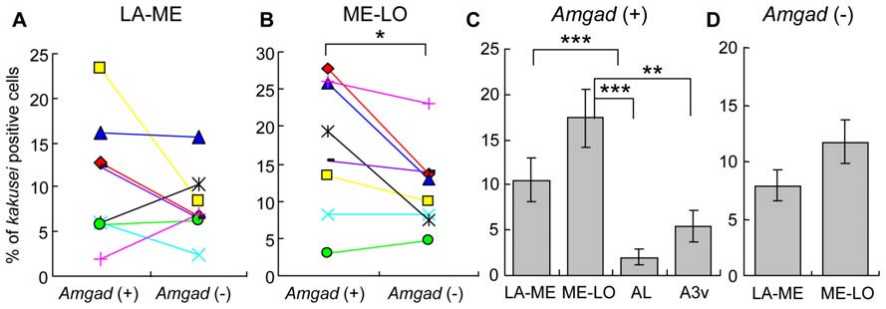
*kakusei* expression in the *Amgad* (+) and (−) cells in the forager brains. The percentages of *kakusei*-positive *Amgad* (+) and (−) cells did not significantly differ in LA-ME (**A**), but did differ in ME-LO (**B**) (*P*<0.05, paired *t*-test). (**C**) The percentage of *kakusei*-positive *Amgad* (+) cells in the optic lobe (LA-ME and ME-LO) was significantly higher than that in AL and A3v (**: *P*<0.02, ***: *P*<0.01; *U*-test). (**D**) There was no significant difference in the *kakusei* expression between LA-ME and ME-LO among the *Amgad* (−) cells.

We then investigated GABAergic neuron activity in the forager brains. *kakusei* was expressed both in *Amgad* (+) and (−) cells in LA-ME and ME-LO (Fig. 3). In LA-ME, no significant difference in the percentage of *kakusei*-positive *Amgad* (+) or (−) cells was detected (Fig. 3A). In contrast, in ME-LO, the percentage of *kakusei*-positive *Amgad* (+) cells was significantly greater than that of *kakusei*-positive *Amgad* (−) cells (*P*<0.05, paired *t*-test; Fig. 3B), suggesting that *kakusei*-related activity of GABAergic neurons was higher than that of non-GABAergic neurons. Among the *Amgad* (+) cells, *kakusei*-expression was significantly higher in LA-ME and ME-LO than in the ALs and A3v, suggesting that *kakusei*-related activity of GABAergic neurons was increased preferentially in the forager OLs (Fig. 3C). In contrast, there was no significant difference between LA-ME and ME-LO in either *Amgad* (+) or (−) cells (Fig. 3C and D).

### *kakusei*-Related Activity of GABAergic Neurons in the Brains of Re-Orienting Bees

**Figure 4:**
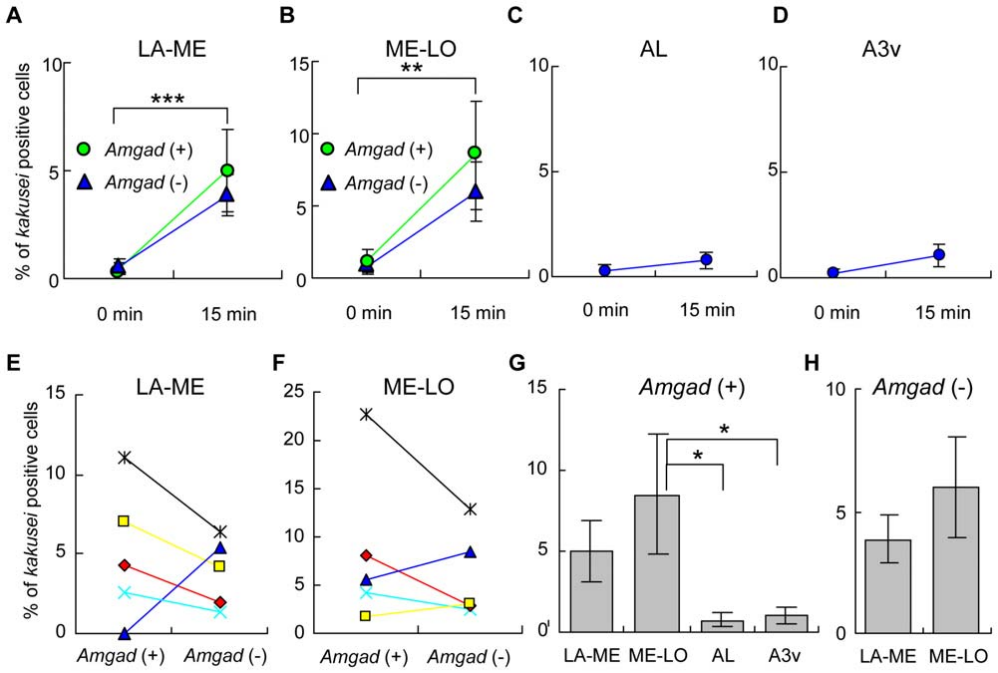
*kakusei* expression in the brains of re-orienting bees. **A–D**. Changes in *kakusei* expression induced by re-orientation. *kakusei* expression was significantly increased both in LA-ME (**A**) and ME-LO (**B**) (**: *P*<0.03, ***: *P*<0.01). The increase was similar between *Amgad* (+) and (−) cells. No significant increase was detected in AL (**C**) or A3v (**D**). The percentages of *kakusei*-positive cells *Amgad* (+) and (−) cells did not differ significantly between LA-ME (**E**) and ME-LO (**F**). (**G**) The percentage of *kakusei*-positive *Amgad* (+) cells in ME-LO was significantly higher than that in AL and A3v (*: *P*<0.02, respectively). (**H**) The percentage of *Amgad* (−) cells did not differ significantly between LA-ME and ME-LO.

In the OLs, especially in the lobula, visual information, which is related to foraging behavior, such as color and image motion, is processed and conveyed to the higher brain centers [27], [28]. Thus, we examined whether the increased *kakusei*-related activity of GABAergic neurons in the forager OLs is related to foraging behavior, such as flight distance estimation [1], or simply to increased visual input during the foraging flights. To examine whether the simple visual experience during flight induces a biased increase in GABAergic neuron activity in the OLs, we investigated *kakusei*-expression in the re-orienting bees, which flew to memorize the new hive location [7]. The proportion of *kakusei*-positive cells in the OLs drastically increased in both *Amgad* (+) and (−) cells after 15 min of re-orienting flight, but no significant difference was observed between *Amgad* (+) and (−) cells (Fig. 4A and B). In contrast, no significant increase was detected in AL or A3v (Fig. 4C and D). The percentages of *kakusei*-positive *Amgad* (+) and (−) cells in LA-ME or ME-LO were not significantly different in the re-orienting bees (Fig. 4E and F), although the percentage of *kakusei*-positive *Amgad* (+) ME-LO cells was significantly higher than that in the other brain regions (Fig. 4G). The percentage of *kakusei*-positive *Amgad* (+) or (−) cells was not significantly different between LA-ME and ME-LO (Fig. 4G and H). These results indicate that GABAergic and non-GABAergic neurons in the OLs are activated in a similar manner by the re-orienting flight, and suggest that increased *kakusei*-related activity of GABAergic neurons in ME-LO is specific to the foragers.

### *kakusei*-Related Activity of GABAergic Neurons in the Brains of Light-Exposed Bees

**Figure 5:**
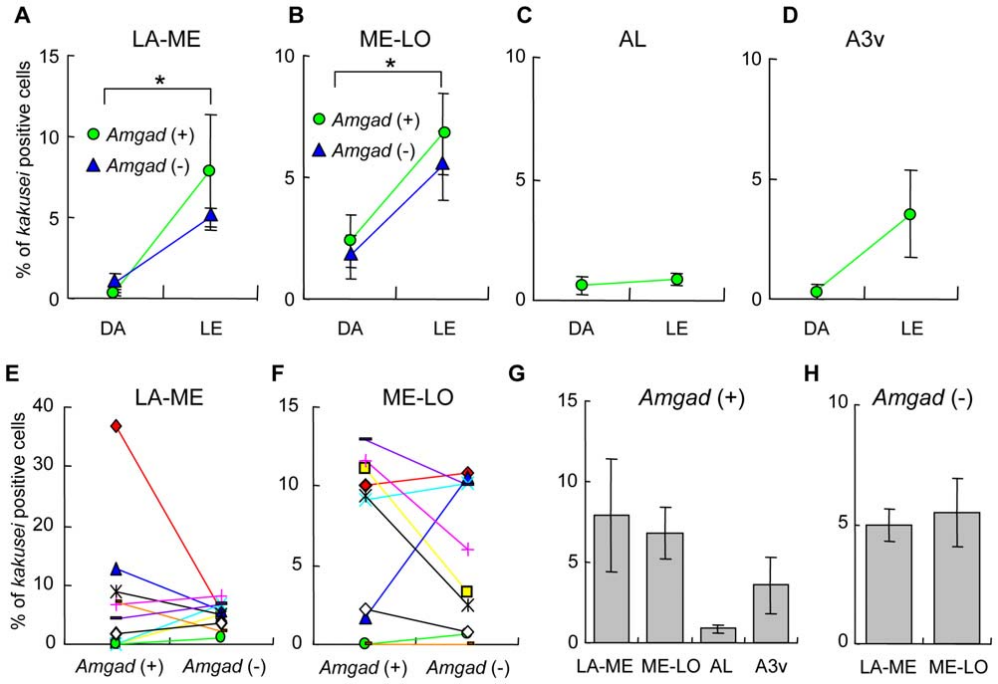
*kakusei* expression in the brains of dark-adapted (DA) and light-exposed (LE) bees. **A–D**. Changes in *kakusei* expression induced by the light-exposure. *kakusei* expression was significantly increased in LA-ME (**A**) and ME-LO (**B**) (*: *P*<0.01, respectively). The increase was similar between *Amgad* (+) and (−) cells. No significant increase in AL (**C**) or A3v (**D**) was detected. The percentages of *kakusei*-positive *Amgad* (+) and (−) cells did not differ significantly in LA-ME (**E**) or ME-LO (**F**). **G**, **H**. The percentage of *kakusei*-positive cells was not significantly different among various brain regions.

In a previous study, we detected strong *kakusei*-expression in OL neurons induced by exposing dark-adapted workers to light [7]. Thus, we next investigated *kakusei*-expression in the brains of light-exposed workers to examine whether the simple light-exposure stimulates GABAergic neuron activity in the OLs. Light exposure preferentially increased *kakusei*-expression both in *Amgad* (+) and (−) cells of the OLs (Fig. 5A and B). In contrast, no significant increase was observed in either AL or A3v (Fig. 5C and D). There was no significant difference in *kakusei*-expression between *Amgad* (+) and (−) cells of the OLs (Fig. 5E and F). In addition, we did not detect any significant difference in the percentage of *kakusei*-positive cells among brain regions (Fig. 5G and H). These results suggest that GABAergic and non-GABAergic neurons in the OLs are activated in the same manner by simple light-exposure.

## Discussion

In the present study, we isolated *Amgad* and revealed GABAergic neuron activity based on simultaneous detection of *Amgad* and *kakusei* expression by double-*in situ* hybridization in the worker brain. Although activity of GABAergic neurons was previously investigated in immobilized workers using electrophysiologic methods [29], this is the first report of the detection of GABAergic neuron activity in free-moving worker honeybees. In the honeybee brain, GABAergic neurons play essential roles in sensory processing like olfaction and in sensory integration like olfactory learning. The GABAergic neurons are heterogeneously distributed to the whole brain area and form no obvious clusters [12]–[14], making it difficult to investigate GABAergic neuronal activity with other methods. Therefore, our results provide the first insight into the functional importance of GABAergic neurons in the brains of free-moving honeybees.

In the brains of forager, re-orienting, and light-exposed bees, we detected high *kakusei*-related activity in GABAergic and non-GABAergic neurons in the OLs, compared with the naïve bees [0 min group of re-orientation bees (Fig. 4A–D) and dark-adapted bees (Fig. 5A–D)], which have almost no *kakusei* expression. The increase in OL neuronal activity is reasonable because these bees are visually stimulated by their behavior or treatment. In contrast, we detected significantly lower *kakusei-*related activity in the AL neurons than in the OL neurons in these bees (Fig. 3C). This is somehow contradictory to previous behavioral studies, however, that showed the importance of both vision and olfaction for navigating and foraging [1], [2], [30], [31]. Why then did we detect low *kakusei*-related neural activity in the ALs? Low *kakusei*-related neural activity in the AL neurons might be due to less frequent or transient AL activation during foraging. Foragers receive odor inputs when they are near and on a flower, but olfactory stimulation might not be strong during the foraging flight compared with the visual information. In this sense, visual information processing may be dominant over olfactory information processing in the forager brains.

Generally, *kakusei*-related neuronal activity in GABAergic and non-GABAergic neurons was similar in the OLs of the free-moving workers, suggesting that GABAergic neurons are as important as non-GABAergic neurons in terms of information processing, such as occurs in lateral inhibition. In contrast, we detected activity in a higher percentage of GABAergic neurons than non-GABAergic neurons in ME-LO of the forager brain. This biased *kakusei*-related neural activity in GABAergic neurons was detected only in the forager, suggesting that the neural mechanisms in forager ME-LO differ from those of the re-orienting bees and light-exposed bees. Considering that foragers utilize visual information to calculate flight distance and to determine direction [1], [2], [32], it is plausible that a particular neural activity pattern is observed in forager OLs. It may be that the forager-specific neural activity pattern in the OLs contributes to their small-type Kenyon cell-preferential neural activity.

A3v neurons are GABAergic feedback neurons that receive inputs from the α lobe of the MBs and project to the calyx of the MBs [14], [26], [33], [34]. Activity of A3v neurons is modulated by pairing an odor with sucrose-reward, suggesting a functional role in sensory information integration [29]. Although we tried to assess the MB activity from A3v neuron activity from this point of view, the activity was not clearly correlated with MB activity, which can be expected based on the findings of our previous paper [7], suggesting that A3v neurons respond to other information from the MBs and are not suitable for monitoring MB activity using our methods.

In the present study, we focused our analysis only on GABAergic neurons as an inhibitory neural system. In addition to GABA, histamine and hyperpolarizing glutamate function as inhibitory neural transmitters [8]–[10]. Especially, histamine is used as a neurotransmitter in the insect photoreceptor neurons [8]. Thus, future analysis of these neuron activity will deepen our understanding of the function of inhibitory neurons in the forager brains.

The present study provides the first evidence that GABAergic neuron activity is relatively high in forager and re-orienting bees. Future analysis of the GABAergic neuron network and function in visual information processing will help to further elucidate the neural basis of the highly sophisticated visual ability of the honeybees.

## Supporting Information

Table S1 The numbers of analyzed bees, sections, and cells.
(0.06 MB DOC)
