# Supplementary material for: Analysis of GABAergic and Non-GABAergic Neuron Activity in the Optic Lobes of the Forager and Re-Orienting Worker Honeybee (Apis mellifera L.)
Source: PLoS One. 2010 Jan 21;5(1):e8833. doi: 10.1371/journal.pone.0008833 (PMC2809111; doi:10.1371/journal.pone.0008833)
Supplement: Table S1 — The numbers of analyzed bees, sections, and cells. (0.06 MB DOC) [file pone.0008833.s001.doc]

Table 1

| Bee type | Brain region | Bee | Section | Average cells |
| --- | --- | --- | --- | --- |
| Sz-induced | LA-ME | 6 | 18 | 660.5 ± 166.2 |
|  | ME-LO | 4 | 7 | 239.0 ± 53.4 |
|  | AL | 5 | 9 | 97.8 ± 17.7 |
|  | A3v | 5 | 13 | 115.8 ± 26.1 |
| Forager | LA-ME | 8 | 15 | 449.8 ± 64.4 |
|  | ME-LO | 8 | 11 | 169.6 ± 27.9 |
|  | AL | 7 | 8 | 54.9 ± 6.1 |
|  | A3v | 7 | 11 | 60.0 ± 13.9 |
| Dark-adapted | LA-ME | 8 | 12 | 359.0 ± 69.2 |
|  | ME-LO | 8 | 11 | 162.0 ± 22.2 |
|  | AL | 8 | 11 | 95.8 ± 12.6 |
|  | A3v | 8 | 9 | 41.8 ± 7.8 |
| Light-exposed | LA-ME | 10 | 19 | 446.8 ± 52.4 |
|  | ME-LO | 10 | 16 | 191.9 ± 20.9 |
|  | AL | 10 | 15 | 97.5 ± 12.9 |
|  | A3v | 8 | 11 | 52.4 ± 13.3 |
| Re-orientation  0 min | LA-ME | 4 | 12 | 711.3 ± 18.2 |
|  | ME-LO | 4 | 11 | 323.8 ± 49.3 |
|  | AL | 4 | 9 | 117.3 ± 20.1 |
|  | A3v | 4 | 8 | 95.0 ± 27.3 |
| Re-orientation  15 min | LA-ME | 5 | 15 | 692.6 ± 15.8 |
|  | ME-LO | 5 | 15 | 340.2 ± 53.6 |
|  | AL | 5 | 13 | 139.8 ± 20.9 |
|  | A3v | 6 | 13 | 96.0 ± 19.9 |
